# Solving a mathematical model integrating unequal-area facilities layout and part scheduling in a cellular manufacturing system by a genetic algorithm

**DOI:** 10.1186/s40064-016-2773-5

**Published:** 2016-08-04

**Authors:** Ahmad Ebrahimi, Reza Kia, Alireza Rashidi Komijan

**Affiliations:** Department of Industrial Engineering, Firoozkooh Branch, Islamic Azad University, Firoozkooh, Iran

**Keywords:** Cellular manufacturing system, Machine layout, Part scheduling, Mixed-integer nonlinear programming, Genetic algorithm

## Abstract

In this article, a novel integrated mixed-integer nonlinear programming model is presented for designing a cellular manufacturing system (CMS) considering machine layout and part scheduling problems simultaneously as interrelated decisions. The integrated CMS model is formulated to incorporate several design features including part due date, material handling time, operation sequence, processing time, an intra-cell layout of unequal-area facilities, and part scheduling. The objective function is to minimize makespan, tardiness penalties, and material handling costs of inter-cell and intra-cell movements. Two numerical examples are solved by the Lingo software to illustrate the results obtained by the incorporated features. In order to assess the effects and importance of integration of machine layout and part scheduling in designing a CMS, two approaches, sequentially and concurrent are investigated and the improvement resulted from a concurrent approach is revealed. Also, due to the NP-hardness of the integrated model, an efficient genetic algorithm is designed. As a consequence, computational results of this study indicate that the best solutions found by GA are better than the solutions found by B&B in much less time for both sequential and concurrent approaches. Moreover, the comparisons between the objective function values (OFVs) obtained by sequential and concurrent approaches demonstrate that the OFV improvement is averagely around 17 % by GA and 14 % by B&B.

## Background

Nowadays in modern competitive manufacturing environments, each company will be required to be capable of reacting quickly to sudden unpredictable changes in a market. Hence, flexibility and efficiency in production have been the main targets of many manufacturing systems such as flexible manufacturing systems (FMS) and just-in-time (JIT) production (Adeil et al. 1996; Selim et al. 1997). One of the major approaches to enhance both flexibility and efficiency is cellular manufacturing (CM), which is an important application of group technology (GT) that handle the formation of manufacturing cells in a way that each part family is processed using a machine cell (Wemmerlov and Hyer 1986). Three major considerable steps in a successful design of a cellular manufacturing system (CMS) are: (1) cell formation (CF) (i.e., to group parts with similar processing requirements into part families and machines to machine cells); (2) group layout (GL) (i.e., to assign machines to workstations within each cell, called intra-cell layout, and cells arrangements within shop floor, called inter-cell layout), and (3) group scheduling (GS) (i.e., scheduling of part families) (Jajodia et al. 1992; Wu et al. 2007b).

The cell formation problem is an area that has been widely investigated in the literature (Soleimanpour et al. 2002). Detailed literature reviews can be found in the cell formation problems’ review (Papaioannou and Wilson 2010). Cellular layout in the CMS design is the subject of some studies that has not received as much attention as cell formation problem in the past two decades (Wang et al. 2001). Most studies assume the first stage (CF) as a priority and then solve the inter-cell and intra-cell layout problems while some others assume the joint problem of CF and GL. Another decision in the CMSs is GS that has been studied by some researchers but only a few studies have attempted to join GS decision with other decisions (i.e., CF and GL). Recently-published articles considering these decisions are investigated and summarized in Table 1.

**Table 1:** Studies integrating CF, machines layout, and parts scheduling decisions in CM design

References	Decisions	Integrating approach	Solution method
Chandrasekharan and Rajagopalan (1993)	CF, GL	Sequential	Non-metric multidimensional scaling
Jajodia et al. (1992)	CF, GL	Sequential	Simulated annealing (SA)
Salum (2000)	CF, GL	Sequential	Two phase method based on manufacturing lead time (MLT)
Urban et al. (2000)	CF, GL	Sequential	Mathematical model based on quadratic assignment problem (QAP) and network flow problem (NFP)
Alfa et al. (1992)	CF, GL	Concurrent	Mathematical model-SA
Bazargan-lari et al. (2000)	CF, GL	Concurrent	Three-phase approach
Wang et al. (2001)	CF, GL	Concurrent	Mathematical model-SA
Arvindh and Irani (1994)	CF, GL	Concurrent	An integrated framework
Akturk (1996)	CF, GL	Concurrent	Mathematical model
Chiang and Lee (2004)	CF, GL	Concurrent	SA
Mahdavi et al. (2008)	CF, GL	Concurrent	Heuristic based on flow matrix
Ahi et al. (2009)	CF, GL	Concurrent	Multiple attribute decision making (MADM)
Wu et al. (2006)	CF, GL	Concurrent	Genetic algorithm (GA)
Wu et al. (2007a)	CF, GL	Concurrent	Genetic algorithm (GA)
Wu et al. (2007b)	CF, GL, GS	Concurrent	Mathematical model–Hierarchical GA (HGA)
Mahdavi and Mahadevan (2008)	CF, GL	Concurrent	Heuristic approach
Solimanpur et al. (2004b)	CF, GL	Concurrent	Mathematical model based on QAP-Ant colony optimization (ACO)
Castillo and Westerlund (2005)	CF, GL	Concurrent	An *e*-accurate model
Jolai et al. (2012)	CF, GL	Concurrent	Electromagnetism algorithm
Xie and Sahinidis (2008)	CF, GL	Concurrent	Branch and bound
Javadi et al. (2013)	CF, GL	Concurrent	Mathematical model
Mohammadi and Forghani (2014)	CF, GL	Concurrent	Genetic algorithm (GA)
Sridhar and Rajendran (1993)	CF, GS	Sequential	Hybrid SA
Solimanpur et al. (2004a)	CF, GS	Sequential	Two-stage heuristic algorithm
Atmani et al. (1995)	CF, GS	Concurrent	Mathematical model
Franca et al. (2005)	CF, GS	Concurrent	Evolutionary algorithm
Reddy and Narendran (2003)	CF, GS	Concurrent	Heuristic
Leung et al. (2007)	CF, GS	Concurrent	Heuristic
Lin et al. (2009)	CF, GS	Concurrent	Tabu search (TS)–GA–SA
Hendizadeh et al. (2008)	CF, GS	Concurrent	Tabu Search (TS)
Tavakkoli-Moghaddam et al. (2008)	CF, GS	Concurrent	GA and Memetic algorithm
Tavakkoli-Moghaddam et al. (2010)	CF, GS	Concurrent	Scatter search (SS)
Chen and Cao (2004)	CF, GS	Concurrent	Mathematical model–TS
Arkat et al. (2012a, b)	CF, GL, GS	Sequential	Mathematical model–GA

Due to the complexity and NP-complete nature of CF, GL, and GS decisions, most researchers have addressed two or three decisions sequentially or independently. However, the benefits gained from CMS implementation are highly affected by how three stages of the CMS design have been performed in collaboration with each other. Hence, all of these decisions should be addressed concurrently with the intention of obtaining the best results (Alfa et al. 1992; Bazargan-lari et al. 2000).

Ranjbar and Najafian Razavi (2012) proposed a new approach to concurrently make the layout and scheduling decisions in a job shop environment and developed a hybrid metaheuristic approach based on the scatter search algorithm. Ripon and Torresen (2013) presented a multi-objective evolutionary method based on a hybrid genetic algorithm by incorporating variable neighborhood search for solving job shop scheduling problem (JSSP) that considers transportation delays and facility layout planning (FLP) as an integrated problem. Halat and Bashirzadeh (2014) developed a concurrent approach for job shop cell scheduling to minimize the makespan in an integer linear programming model by considering exceptional elements, intercellular moves, intercellular transportation times, and sequence-dependent family setup times. They also developed a heuristic approach based on the genetic algorithm.

Wu et al. (2007b) have extended the mathematical models proposed in Wu et al. (2006, 2007a) to develop a new one which integrated three mentioned decisions. Then, they developed a hierarchical genetic algorithm (HGA) to solve the integrated cell design problem. The deficiencies of that model are inaccuracy in determining the layout of cells and probable overlapping of the cells. Tang et al. (2010) developed a scatter search approach to solve a nonlinear mathematical programming model for the problem of parts scheduling in a CMS by considering exceptional parts by minimizing the total weighted tardiness.

Arkat et al. (2012a) have promoted a similar integration by proposing two mathematical models, the first one integrates cellular layout with cell formation to determine optimal cell configuration and the layout of machines and cells in order to minimize the total movement costs. Considering the results obtained by solving this model integrating cellular configuration and layout, the cell scheduling problem becomes a job shop scheduling problem with transportation times. The second model is based on the concurrent design and integrates the GS problem with CF and GL problems. Also, two genetic algorithms were developed to solve the real-sized problems. Arkat et al. (2012b) presented a multi-objective model to make decisions about cell formation, cellular layout and operation sequence simultaneously. The first objective was to minimize total transportation cost of parts and the second objective was to minimize makespan. A multi-objective genetic algorithm was used to solve the model. Zeng et al. (2015) proposed a two-stage GA-based heuristic algorithm to solve a nonlinear mathematical programming model to determine the sequences of the exceptional parts to be transferred via an automated guided vehicle (AGV) in order to minimize the process make-span.

As the main aim of this article, regarding the articles reviewed above, is proposing a new mathematical model with consisting of important manufacturing features such as operation sequence, processing time, transferring time, intra-cell layout and parts scheduling. The presented model is different from the existing models available in the literature because of incorporating some important design aspects simultaneously. In the first aspect, the layout of machines with unequal-areas in the cells is not restricted to linear type. However, dimensions of cells are predetermined by a system designer in a shop floor with a continuous area. In the second aspect, process routings for part types can be flexible. In the third aspect, time and cost of movement are depended on three factors: movement distance, part type, and movement type (inter-cell or intra-cell). In the fourth aspect, despite the fact that three ingredients have been formulated in the objective function including makespan, penalty cost, and material handling costs, however, only one, two, or three of them can be used to form cells based on the desired objective. Finally, in the fifth aspect, the CF, machines layout, and parts scheduling decisions are made simultaneously by an integrated model.

Another aim of this article is developing an efficient genetic algorithm enhanced by a matrix-based chromosome structure consisting of two sections for layout and scheduling, a heuristic procedure generating initial feasible solutions, a procedure calculating the fitness functions of generated solutions, and efficient crossover and mutation operators in order to determine three interrelated stages in designing a CMS simultaneously.

The remainder of this article is organized as follows. In “Mathematical model” section, a mathematical model integrating CMS, machines layout and parts scheduling decisions is formulated. The development of the designed GA is discussed in “Designed genetic algorithm for the integrated layout and scheduling model of a CMS” section. “Experimental results” section illustrates the test problems that are utilized to investigate the features of the proposed model and the performance of the developed algorithm. Finally, a conclusion is given in “Conclusion” section.

## Mathematical model

### Model assumptions

In this section, a mathematical model is formulated to minimize three ingredients in the objective function including makespan, tardiness penalty, and inter-cell and intra-cell material handling costs under the following assumptions:

1. Each part type has several operations which should be processed in a given sequence. Also, the processing capabilities and processing times of part-operations for each machine type are known and deterministic.
2. All machine types are assumed to be multi-purpose ones which are capable of performing one or more operations without imposing a reinstalling cost. In like manner, each operation of a part type can be performed on different machine types with different processing times. This feature providing flexibility to process plan of parts has been known as alternative process routings. Nevertheless, a part operation should be processed by only one of those machines which are capable of processing that operation.
3. A machine cannot process more than one part at the same time.
4. Parts are moved individually by material handling devices between and within cells. Inter-cell movement happens whenever successive operations of a part type are carried out in different cells. Also, the intra-cell movement happens whenever successive operations of a part type are processed on different machines in the same cell.
5. The rectangular facilities of unequal-areas can be located anywhere in the cells having a predetermined shape with a continual space without any overlaps. In fact, the inter-cell layout and distances between cells are given and intra-cell layout is determined by the model.
6. Each planar cell has a rectangular shape whose length and width is known in advance. Also, the number of cells to be formed is given.
7. The maximum and minimum limit of the cell size in terms of the number of machines is known.
8. The loading and unloading point is at the center of each machine.
9. Machines have a predetermined orientation (i.e., machines may be located either horizontally or vertically). The machine is horizontally located if the longer side of the machine is parallel to the x-axis. On the contrary, the machine is vertically located if the longer side is parallel to the y-axis.
10. The rectilinear distance between the centers of two facilities *i* and *j* with coordinates (*x*_*i*_, *y*_*i*_) and (*x*_*j*_, *y*_*j*_) is considered to be the distance norm: *d*_*ij*_ = |*x*_*i*_ − *x*_*j*_| + |*y*_*i*_ − *y*_*j*_|.
11. Once an operation of a part starts to be processed on a machine, it cannot be interrupted before being completed.
12. Due date is determined for each part. As a result, tardiness penalty is incurred for each part type per time unit if it is not completed before its due date.
13. Cost and time of handling a part between two locations in the same cell or between different cells depend on three factors: the distance between locations of machines, type of part, and type of movement (inter-cell or intra-cell). Hence, for each part type, three coefficients per distance unit are considered: movement time, inter-cell movement cost and intra-cell movement cost.
14. Machine setup time is negligible.
15. The machines will never breakdown and be available throughout the scheduling period.

The notations used in the model are presented below:

### Sets

$$i = \left\{ {1,2, \ldots ,P} \right\}$$
index of parts
$$j,j^{{\prime }} = \left\{ {1,2, \ldots ,M} \right\}$$
index of machines
$$c,c^{{\prime }} = \left\{ {1,2, \ldots ,C} \right\}$$
index of cells
$$k = \left\{ {1,2, \ldots ,K_{p} } \right\}$$
index of operations for part type *p*
$$k^{\prime},k^{{\prime \prime }} = \left\{ {1,2, \ldots ,K_{m} } \right\}$$
index of processing positions for machine type *m*

### Model parameters

$$Q$$
factory costs per time unit
$$Ply_{i}$$
tardiness penalty for part type *i* per time unit
$$dd_{i}$$
due date for part type *i*
$$CO_{i}$$
inter-cell material handling cost for part type *i* per distance unit
$$CI_{i}$$
intra-cell material handling cost for part type *i* per distance unit
$$TT_{i}$$
material handling time for part type *i* per distance unit
$$UBC$$
upper cell size limit
$$LBC$$
lower cell size limit
$$T_{kij}$$
processing time of operation *k* of part type *i* on machine *j*
$$a_{kij}$$
1 if operation *k* of part type *i* can be processed on machine type *j*; 0 otherwise (i.e., $a_{kij}$ is 1 if $T_{kij} > 0$; 0 otherwise)
$$L_{j}$$
length of the horizontal side of machine type *j*
$$H_{j}$$
height of the vertical side of machine type *j*
$$LX_{c}$$
horizontal coordinate of left side of cell *c*
$$RX_{c}$$
horizontal coordinate of right side of cell *c*
$$LY_{c}$$
vertical coordinate of lower side of cell *c*
$$UY_{c}$$
vertical coordinate of upper side of cell *c*
$$M$$
a big positive number

### Decision variables

$$V_{jc}$$
1 if machine *j* is assigned to cell *c*,0 otherwise
$$Z_{ki}^{k'j}$$
1 if *k*th operation of part type *i* is processed at *k′*th processing position on machine *j*, 0 otherwise
$$CTM_{k'j}$$
completion time of *k′* th processing position of machine *j*
$$CTP_{i}$$
completion time of part type *i*
$$C_{\rm{max} }$$
makespan time
$$\alpha_{j}$$
horizontal coordinate of center of machine *j*
$$\beta_{j}$$
vertical coordinate of center of machine *j*
$$x_{j}^{{\prime }}$$
horizontal coordinate of left side of machine *j*
$$x_{j}^{{\prime \prime }}$$
horizontal coordinate of right side of machine *j*
$$y_{j}^{{\prime }}$$
vertical coordinate of lower side of machine *j*
$$y_{j}^{{\prime \prime }}$$
vertical coordinate of upper side of machine *j*
$$R_{jj'}^{X}$$
1 if machine *j* is completely located right side of machine *j′*, 0 otherwise
$$R_{jj'}^{Y}$$
1 if machine *j* is completely located upper side of machine *j′*, 0 otherwise
$$d_{jj'}$$
distance between machine *j* and *j′*

### Mathematical model

 The mix-integer nonlinear mathematical model is presented as follow:

*Minimize*

1.1 $$Z = Q \times C_{\hbox{max} }$$

1.2 $$+ \mathop \sum \limits_{i = 1}^{P} \left( {Ply_{i} \times \hbox{max} \left\{ {0 , CTP_{i} - dd_{i} } \right\}} \right)$$

1.3 $$+ \mathop \sum \limits_{k = 2}^{{K_{p} }} \mathop \sum \limits_{i = 1}^{P} \mathop \sum \limits_{j = 1}^{M} \mathop \sum \limits_{{j^{\prime} \ne j}} \mathop \sum \limits_{c = 1}^{C} \left( {\left( {\mathop \sum \limits_{{k^{\prime} = 1}}^{{K_{m} }} Z_{ki}^{{k^{\prime}j}} } \right) \times \left( {\mathop \sum \limits_{{k^{\prime} = 1}}^{{K_{m} }} Z_{k - 1i}^{{k^{\prime}j^{\prime}}} } \right) \times \left[ {\left( {V_{jc} \times V_{j'c} \times CI_{i} } \right) + \left( {V_{jc} \times \left( {1 - V_{{j^{\prime}c}} } \right) \times CO_{i} } \right)} \right] \times d_{jj'} } \right)$$

*Subject to:*

2 $$\sum\limits_{c = 1}^{C} {V_{jc} } = 1\quad \forall j$$

3 $$\mathop \sum \limits_{j = 1}^{M} V_{jc} \le UBC\quad \forall c$$

4 $$\mathop \sum \limits_{j = 1}^{M} V_{jc} \ge LBC\quad \forall c$$

5 $$\mathop \sum \limits_{{k^{\prime} = 1}}^{{K_{m} }} \mathop \sum \limits_{j = 1}^{M} Z_{k i}^{k'j} = 1\quad \forall \;k,i$$

6 $$\mathop \sum \limits_{{k^{\prime} = 1}}^{{K_{m} }} Z_{ki}^{{k^{\prime}j}} \le a_{kij} \quad \forall k,i,j$$

7 $$CTM_{1j} = \mathop \sum \limits_{i = 1}^{P} \left( {\mathop \sum \limits_{k = 2}^{{K_{p} }} Z_{ki}^{1j} \times \left[ {T_{kij} + \mathop \sum \limits_{{k^{\prime \prime } = 1}}^{{K_{m} }} \mathop \sum \limits_{{j^{\prime } = 1}}^{M} Z_{k - 1i}^{{k^{\prime \prime } j^{\prime } }} \left( {CTM_{{k^{\prime \prime } j^{\prime } }} + \left( {d_{{jj^{\prime } }} \times TT_{i} } \right)} \right)} \right] + Z_{1i}^{1j} \cdot T_{1ij} } \right)\quad \forall j$$

8 $$CTM_{{k^{\prime } j}} = \mathop \sum \limits_{i = 1}^{P} \left( { \mathop \sum \limits_{k = 2}^{{K_{p} }} Z_{ki}^{{k^{\prime } j}} \times \left[ { T_{kij} + {\text{Max}}\left\{ {CTM_{{k^{\prime} - 1j}} ,\quad \mathop \sum \limits_{{k^{\prime \prime } = 1}}^{{K_{m} }} \mathop \sum \limits_{{j^{\prime} = 1}}^{M} Z_{k - 1i}^{{k^{\prime \prime } j^{{\prime }} }} \left( {CTM_{{k^{\prime \prime } j^{{\prime }} }} + \left( {d_{{jj^{\prime}}} \times TT_{i} } \right)} \right)} \right\}} \right] + Z_{1i}^{{k^{\prime } j}} \times \left[ {T_{1ij} + CTM_{{k^{\prime} - 1j}} } \right] } \right)\quad \forall k^{\prime} > 1, j$$

9 $$CTP_{i} = \mathop \sum \limits_{{k^{\prime} = 1}}^{{K_{m} }} \mathop \sum \limits_{j = 1}^{M} Z_{kp i}^{{k^{\prime}j}} \cdot CTM_{{k^{\prime}j}} \quad \forall i$$

10 $$C_{\hbox{max} } = Max\left\{ {\forall i:CTP_{i} } \right\}$$

11 $$\alpha_{j} = \frac{1}{2}\left( {x_{j}^{\prime } + x_{j}^{\prime \prime } } \right)\quad \forall j$$

12 $$\beta_{j} = \frac{1}{2}\left( {y_{j}^{\prime } + y_{j}^{\prime \prime } } \right)\quad \forall j$$

13 $$x_{j}^{{\prime \prime }} - x_{j}^{{\prime }} = L_{j} \quad \forall j$$

14 $$y_{j}^{{\prime \prime }} - y_{j}^{{\prime }} = H_{j} \quad \forall j$$

15 $$d_{jj'} = \left| {\alpha_{j} - \alpha_{j'} } \right| + \left| {\beta_{j} - \beta_{j'} } \right|\quad \forall j,j^{\prime} \quad and\quad j \ne j^{{\prime }}$$

16 $$x_{j}^{{\prime \prime }} \le RX_{c} + \left( {1 - V_{jc} } \right)M\quad \forall j,c$$

17 $$LX_{c} \le x_{j}^{{\prime }} + \left( {1 - V_{jc} } \right)M\quad \forall j,c$$

18 $$y_{j}^{{\prime \prime }} \le UY_{c} + \left( {1 - V_{jc} } \right)M\quad \forall j,c$$

19 $$LY_{c} \le y_{j}^{{\prime }} + \left( {1 - V_{jc} } \right)M\quad \forall j,c$$

20 $$R_{{jj^{{\prime }} }}^{X} + R_{{j^{{\prime }} j}}^{X} + R_{{jj^{{\prime }} }}^{Y} + R_{{j^{{\prime }} j}}^{Y} \ge 1\quad \forall j,j^{\prime}\quad and \quad j \ne j^{\prime}$$

21 $$x_{j'}^{{\prime \prime }} \le x_{j}^{{\prime }} + \left( {1 - R_{jj'}^{X} } \right)M\quad \forall j,j^{\prime}\quad and \quad j \ne j^{\prime}$$

22 $$x_{j}^{{\prime \prime }} \le x_{{j^{{\prime }} }}^{{\prime }} + \left( {1 - R_{{j^{{\prime }} j}}^{X} } \right)M\quad \forall j,j^{\prime}\quad and \quad j \ne j^{\prime}$$

23 $$y_{{j^{\prime}}}^{{\prime \prime }} \le y_{j}^{{\prime }} + \left( {1 - R_{{jj^{{\prime }} }}^{Y} } \right)M\quad \forall j,j^{\prime}\quad and \quad j \ne j^{\prime}$$

24 $$y_{j}^{{\prime \prime }} \le y_{{j^{\prime}}}^{{\prime }} + \left( {1 - R_{{j^{{\prime }} j}}^{Y} } \right)M\quad \forall j,j^{\prime}\quad and \quad j \ne j^{\prime}$$

25 $$Z_{ki}^{{k{\prime }j}} ,V_{j} \;is\;Binary\quad \forall k,i,k^{\prime},j$$

26 $$CTM_{{k^{\prime}j}} ,CTP_{i} ,C_{\hbox{max} } \ge 0\quad \forall k^{\prime},j$$

27 $$d_{{jj^{\prime } }} ,\alpha_{j} ,\beta_{j} ,x_{j}^{\prime } ,x_{j}^{\prime \prime } ,y_{j}^{\prime } ,y_{j}^{\prime \prime } \ge 0 \quad \forall j,j^{\prime} j \ne j'$$

28 $$LX_{c} ,UX_{c} ,LY_{c} ,UY_{c} \ge 0\quad \forall c$$

29 $$R_{{jj^{\prime } }}^{X} ,R_{{jj^{\prime } }}^{Y} \;is\;Binary\quad \forall j,j^{\prime}\quad j \ne j'$$

The objective function consists of three terms. Term (1.1) contains makespan time and calculates factory costs during makespan time. Term (1.2) is total tardiness penalties for all parts which have been completed after their due date. Terms (1.3) calculates the inter-cell and intra-cell material handling costs.

Constraints sets (2) ensure that each machine is assigned to only one cell. The cell size limits specified by a designer are enforced through Constraints (3) and (4). Constraint set (5) ensures that each operation of each part is processed by only one machine at one of its processing positions. Constraint set (6) ensures an operation of a part is processed by a machine at one of its processing positions provided that machine is capable of processing the related operation.

Constraints (7) compute completion time of the first processing position of a machine in which an operation of a part is processed. There are two cases: (1) the first operation of part *i*is processed at the first processing position of machine *j*. It can be simply understood that completion time of the first processing position of machine *j*, in this case, is equal to the processing time of the first operation of part *i*; (2) any operation of part *i* except the first one is processed at the first processing position of machine *j*. In this case, the part needs to be moved from the machine *j′* processing the previous operation (i.e., *k* − 1) to the current machine *j* processing the current operation (i.e., *k*). Considering the distance between machines *j* and *j′*, this movement needs a handling time equal to $\left( {d_{{jj^{\prime}}} \times TT_{i} } \right)$. Since the previous operation *k* − 1 of part *i* has been finished at the time $CTM_{{k^{{\prime \prime }} j^{{\prime }} }}$, the part *i* will be ready for processing the operation *k* on machine *j* at time $\left( {d_{{jj^{{\prime }} }} \times TT_{i} } \right) + CTM_{{k^{{\prime \prime }} j^{{\prime }} }}$. As a result, in this case, the completion time of the first processing position of machine *j* is when processing the operation *k* of part *i* on machine *j* is finished and it is equal to $T_{kij} + \left( {d_{{jj^{{\prime }} }} \times TT_{i} } \right) + CTM_{{k^{{\prime \prime }} j^{{\prime }} }}$.

Constraints (8) compute completion time of any processing position except the first one of machine *j* in which an operation of a part is processed. Similarly, there are two cases: (1) the first operation of part *i* is processed at the processing position *k′* of machine *j*. It can be simply understood that completion time of the processing position *k′* of machine *j*, in this case, is equal to processing time of the first operation of part *i* on machine *j* plus the completion time of the previous processing position *k′* − 1 of machine *j*; (2) any operation of part *i* except the first one is processed at the processing position *k* of machine *j*. In this case, as it was similarly explained for Constraint (7), the ready time to processes the operation *k* of part *i* on machine *j* is $\left( {d_{{jj^{{\prime }} }} \times TT_{i} } \right) + CTM_{{k^{{\prime \prime }} j^{{\prime }} }}$. In addition, machine *j* should be idle to process that operation at its processing position *k′*. It means that the completion of the previous processing position *k′* − 1 of machine *j* should have been reached. To satisfy the limitations of part readiness and machine idleness, the time which is equal to the maximum of $CTM_{{k^{\prime} - 1 j}}$ and $\left( {d_{{jj^{{\prime }} }} \times TT_{i} } \right) + CTM_{{k^{{\prime \prime }} j^{{\prime }} }}$ is considered as an actual starting time to process that operation of part *i*. As a result, the completion time of the processing position *k′* of machine *j* in which operation *k* of part *j* is processed is equal to $T_{kij} + {\text{Max}}\left\{ {CTM_{{k^{\prime} - 1 j}} ,\sum\nolimits_{{k^{\prime \prime } = 1}}^{{K_{m} }} {} \sum\nolimits_{{j^{\prime} = 1}}^{M} {Z_{k - 1i}^{{k^{\prime \prime } j^{\prime } }} \left( {CTM_{{k^{\prime \prime } j^{\prime } }} + \left( {d_{{jj^{\prime}}} \times TT_{i} } \right)} \right)} } \right\} .$ Constraints (7) and (8) also enforce a machine to not process more than one part at the same time.

Constraint set (9) computes completion time for each part. Constraint set (10) returns makespan time based on the computed completion times of all parts.

Constraints (11) and (12) represent the horizontal and vertical coordinates of the center of each machine, respectively. In the other hand, Constraint sets (13) and (14) return the coordinates of four sides of a machine based on its center coordinates and its length and height. Constraint set (15) computes the rectilinear distance between the centers of two machines *j* and *j′*.

Constraint sets (16)–(19) ensure that machines assigned to a cell are placed with regard to coordinates of cell sides (vertically and horizontally). Constraint sets (20)–(24) ensure that machines do not overlap in the horizontal and vertical direction simultaneously. Finally, Constraint sets (25)–(29) are the logical binary and non-negativity necessities on the binary and positive continuous decision variables.

### Linearization techniques

The proposed model is nonlinear in both objective function and constraints sets. Hence, some linearization techniques are proposed to convert the nonlinear model into a linearized counterpart as follows:

The non-line function *Max* in the Eq. (1.2) of objective function can be linearized usingthe following transformation $trdns_{i} = \hbox{max} \left\{ {0 , CTP_{i} - dd_{i} } \right\}$ under the below set of constraints:

30 $$trdns_{i} \ge 0\quad \forall i$$

31 $$trdns_{i} \ge CTP_{i} - dd_{i} \quad \forall i$$

Linearization of function *Max* in Eqs. (8) and (10) in constraint sets is exactly similar to that of Eq. (1.2).

The nonlinear absolute components in constraint set (15) can be linearized using the following transformations $\left| {\alpha_{j} - \alpha_{{j^{{\prime }} }} } \right| = \alpha_{{jj^{\prime}}}^{ + } + \alpha_{{jj^{\prime}}}^{ - }$ and $\left| {\beta_{j} - \beta_{{j^{{\prime }} }} } \right| = \beta_{{jj^{\prime}}}^{ + } + \beta_{{jj^{\prime}}}^{ - }$ under the below set of equations:

32 $$\alpha_{j} - \alpha_{{j^{\prime}}} = \alpha_{{jj^{\prime}}}^{ + } - \alpha_{{jj^{\prime}}}^{ - } \quad \forall j,j^{{\prime }}$$

33 $$\beta_{j} - \beta_{{j^{\prime}}} = \beta_{{jj^{\prime}}}^{ + } - \beta_{{jj^{\prime}}}^{ - } \quad \forall j,j^{{\prime }}$$

34 $$\alpha_{{jj^{{\prime }} }}^{ + } , \alpha_{{jj^{\prime}}}^{ - } \beta_{{jj^{\prime}}}^{ + } ,\beta_{{jj^{\prime}}}^{ - } \ge 0\quad \forall j,j^{{\prime }}$$

There are some product terms between binary variables in Eq. (1.3) which make the model non-linear. For instance, let us introduce a new binary variable $ZZ_{{kik^{{\prime }} jj^{{\prime }} }}$ which equals to multiplying both binary summations $\sum\nolimits_{{k^{{\prime }} }} {Z_{ki}^{{k^{{\prime }} j}} }$ and $\sum\nolimits_{{k^{\prime } }} {Z_{k - 1i}^{{k^{\prime } j}} }$ as given in Eq. (1.3) of the objective function. This nonlinear component can be linearized under the following constraint sets:

35 $$ZZ_{{kijj^{{\prime }} }} \ge \mathop \sum \limits_{{k^{{\prime }} }} Z_{ki}^{{k^{{\prime }} j}} + \mathop \sum \limits_{{k^{{\prime }} }} Z_{k - 1i}^{{k^{{\prime }} j^{{\prime }} }} - 1\quad \forall k,i,k^{\prime},j,j^{\prime}\left( {j \ne j^{{\prime }} } \right)$$

36 $$ZZ_{{kijj^{\prime}}} \le \mathop \sum \limits_{{k^{\prime}}} Z_{ki}^{{k^{\prime}j}} \forall k,i,k^{\prime},j,j^{\prime}\left( {j \ne j^{{\prime }} } \right)$$

37 $$ZZ_{{kijj^{{\prime }} }} \le \mathop \sum \limits_{{k^{{\prime }} }} Z_{k - 1i}^{{k^{{\prime }} j^{{\prime }} }} \quad \forall k,i,k^{\prime},j,j^{\prime}\left( {j \ne j^{{\prime }} } \right)$$

Linearization of the other nonlinear terms multiplying binary variables in Eq. (1.3) is similarly done as explained at the above.

There are still some nonlinear terms in the model multiplying binary variables by continuous variables in Eqs. (7)–(9). For example, to linearize the product term $CTM_{{k^{\prime \prime } j^{\prime } }} \cdot Z_{k - 1i}^{{k^{\prime \prime } j^{\prime } }}$ in Eq. (7), a new positive continuous variable $ZCTM_{{kik^{{\prime \prime }} j^{{\prime }} }}$ which equals to $CTM_{{k^{{\prime \prime }} j^{{\prime }} }} \cdot Z_{k - 1i}^{{k^{{\prime \prime }} j^{{\prime }} }}$ is introduced and the product term can be linearized under the following constraint sets:

38 $$ZCTM_{{kik^{{\prime \prime }} j^{{\prime }} }} \ge CTM_{{k^{{\prime \prime }} j^{{\prime }} }} - M \cdot \left( {1 - Z_{k - 1i}^{{k^{{\prime \prime }} j^{{\prime }} }} } \right)\quad \forall k,i,k^{\prime\prime},j,j^{\prime}\left( {j \ne j^{{\prime }} } \right)$$

39 $$ZCTM_{{kik^{{\prime \prime }} j^{{\prime }} }} \le CTM_{{k^{{\prime \prime }} j^{{\prime }} }} \quad \forall k,i,k^{\prime\prime},j,j^{\prime}\left( {j \ne j^{{\prime }} } \right)$$

40 $$ZCTM_{{kik^{{\prime \prime }} j^{{\prime }} }} \le M \cdot Z_{k - 1i}^{{k^{{\prime \prime }} j^{{\prime }} }} \quad \forall k,i,k^{\prime\prime},j,j^{\prime}\left( {j \ne j^{{\prime }} } \right)$$

Linearization of the other nonlinear terms multiplying binary variables by continuous variables in Eqs. (7)–(9) is similarly done.

## Designed genetic algorithm for the integrated layout and scheduling model of a CMS

The GA is an evolutionary search and optimization technique considering the design process as an evolutionary one. It seeks to find the best solution by generating a population of candidate individuals as the current parents. Using a selection mechanism, crossover and mutation operators, solutions (i.e., offsprings) with more fitness values are expected to be generated from the initial population of parents during successive generations. These generations continue until the algorithm finds an acceptable good solution or meets a terminating condition. Genetic algorithms have been implemented in a wide variety of engineering optimization applications (Gen and Cheng 1997; Man et al. 1999), including cellular manufacturing systems (Shiyas and Pillai 2014; Deljoo et al. 2010; Defersha and Chen 2008; Wu et al. 2007a, b; Kia et al. 2014; Vin and Delchambre 2014).

In this section, a genetic algorithm for solving the integrated layout and scheduling model of a CMS is employed. Principle factors for designing the employed GA are described as follows.

### Chromosome structure

**Fig. 1:**
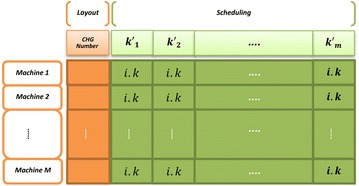
Chromosome structure

As represented in the Fig. 1, the structure of each chromosome is a multi-string, where the number of strings is equal to the number of existing machines and the length of each chromosome stringis $K_{m}^{{\prime }} + 1$. There are two separate sections of information including (1) layout gene and, (2) $K_{m}^{{\prime }}$ schedulinggenes.

In the first section, each layout gene shows an integer number of set $S_{j} = \left\{ {1,2, \ldots ,TS_{j} } \right\}$ determining the coordinate location of each machine. The definition of set $S_{j}$ is discussed at below.

**Fig. 2:**
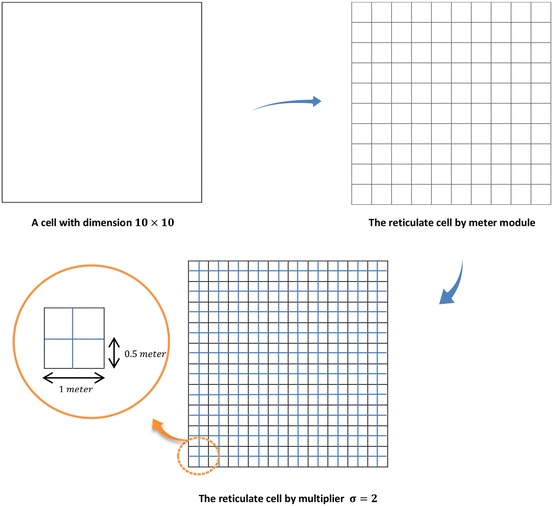
Dividing a cell space into grid cells

The existing cells are divided according to the constant values of length and width of square grids that contain square houses named as Cell Houses (CH’s) and are defined based on a multiplier of the length and width unit. Also, these CH’s are divided into smaller grids named as Grid Cells (GC’s). For example, if the length and width of cells are measured in the meter and the division multiplier is $\sigma = 2$, it means the cells are divided into $0.5 \times 0.5$ CH’s. As another example, if there is a cell with the dimension of $10 \times 10$ m and a multiplier $\sigma = 2$, a grilled cell is obtained as shown in Fig. 2.

The total number of CH’s in a system with *C* cells is equal to:

41 $$TCH_{C} = \mathop \sum \limits_{c = 1}^{C} \left( {UX_{c} - LX_{c} } \right)\left( {UY_{c} - LY_{c} } \right)\sigma^{2}$$

For example, in the above sample having a cell with dimension of $10 \times 10$ m, the total number of CH’s is equal to $TCH_{1} = 100 \times 2^{2} = 400$.

Now, by considering constant length and width values of machines, each machine can be located in finite positions. Each position involves a group of CH’s named as Cell Houses Groups (CHG’s). The entire positions that a machine *j* can be located in CG’s (CHG’s) are defined in set $S_{j} = \left\{ {1,2, \ldots ,TS_{j} } \right\}$, where the total number of positions is calculated as follows:

42 $$TS_{j} = \mathop \sum \limits_{c = 1}^{C} \left[ {\left( {UX_{c} - LX_{c} - L_{j} } \right)\sigma + 1} \right]\left[ {\left( {UY_{c} - LY_{c} - W_{j} } \right)\sigma + 1} \right]$$

CH’s and CHG’s are numbered from the left-down corner to the right-up corner according to the cell numbers. After numbering a cell, the continuous numbering CH’s and CHG’s starts from the next cell and is continued until the last cell.

For example, if there are two cells with dimension of $10 \times 10$ m and $8 \times 10$  m and a $4 \times 2$ m machine *j*, the total number of CH’s and candidate locations in set *S* for this machine are calculated as follows:

$$TCH_{2} = \left( {100 \times 4} \right) + \left( {80 \times 4} \right) = 720$$

$$TS_{j} = \left( {13 \times 17} \right) + \left( {9 \times 17} \right) = 374$$

$$S_{j} = \left\{ {1,2, \ldots ,374} \right\}$$

**Fig. 3:**
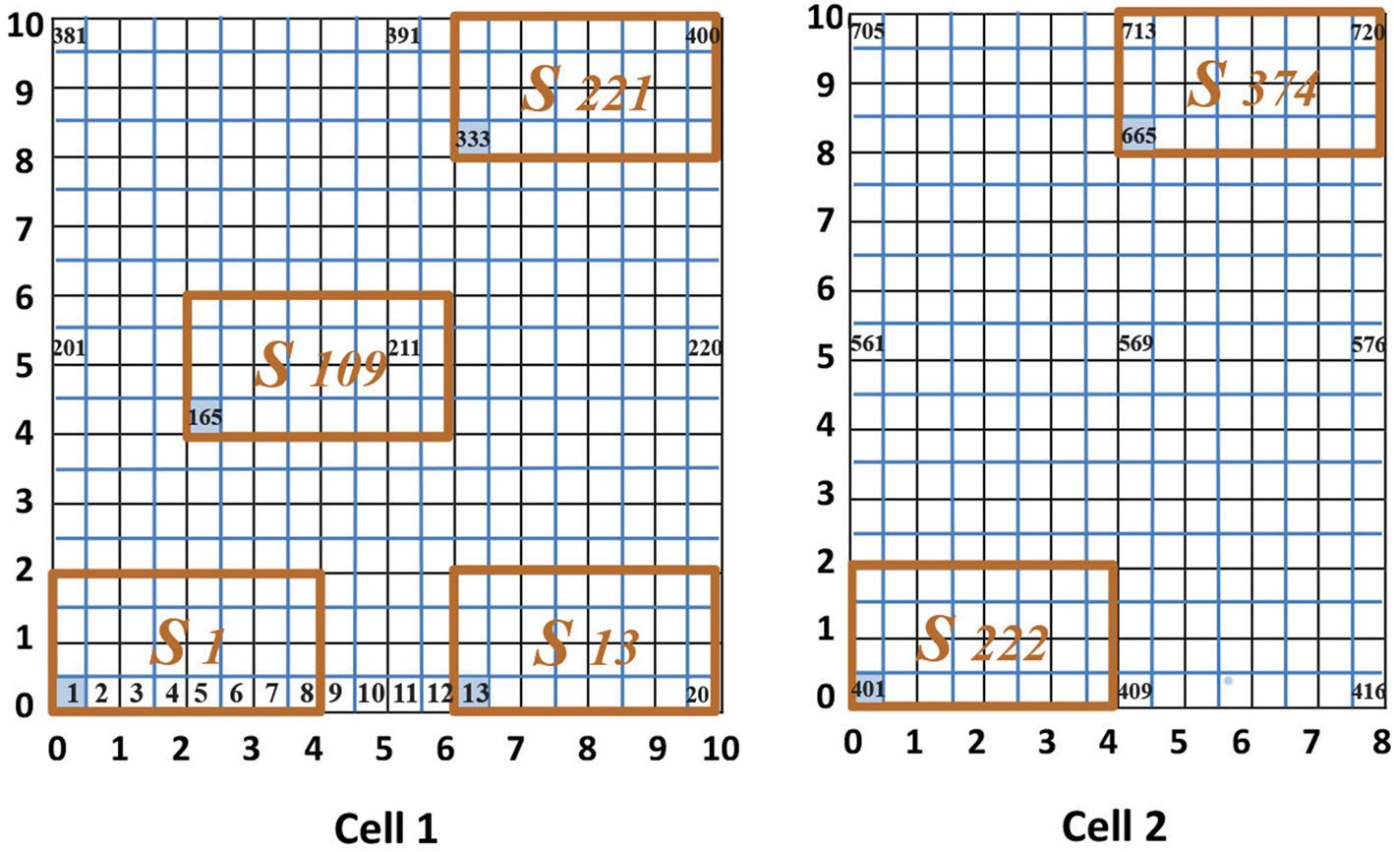
Numbering CHG’s

**Fig. 4:**
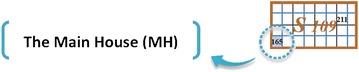
MH of set S_109_

As it was mentioned earlier, each CHG’s contains a set of CH’s. For instance, in the example shown in Fig. 3, the set S_109_ contains CH’s including {165, 166, 167, 168, 169, 170, 171, 172, 185, 186, 187, 188, 189, 190, 191, 192, 205, 206, 207, 208, 209, 210, 211, 212, 225, 226, 227, 228, 229, 230, 231, 232}. Among these CH’s, the first house in the left-down corner is considered as a Main House (MH) of each set of CH’s (Fig. 4).

The scheduling genes in the second section of each chromosome string are order-based permutation of part operation. In this section, the value in each gene contains information related to part number (*i*) and part operation (*k*) that is shown as a decimal number (*i*.*k*) without having any mathematical value. The first number represents part number and the second one shows part operation.

In the following, the calculation formulas for defined parameters in chromosome structure are discussed:

The total number of CH’s for *C* cells is equal to:

43 $$TCH_{C} = \mathop \sum \limits_{c = 1}^{C} \left( {UX_{c} - LX_{c} } \right)\left( {UY_{c} - LY_{c} } \right)\sigma^{2}$$

The number of locations in a set of CHG’s for machine *j* is

44 $$TS_{j} = \mathop \sum \limits_{c = 1}^{C} \left[ {\left( {UX_{c} - LX_{c} - L_{j} } \right)\sigma + 1} \right]\left[ {\left( {UY_{c} - LY_{c} - W_{j} } \right)\sigma + 1} \right]$$

45 $$S_{j} = \left\{ {1,2, \ldots ,TS_{j} } \right\}$$

There are two cases to calculate the cell number where machine *j* is located based on CHG number of machine *j*. In the first case, the cell number where machine *j* is located is one $\left( {c = 1} \right)$ if:

46 $$CHG\;number \le \left[ {\left( {UX_{1} - LX_{1} - L_{j} } \right)\sigma + 1} \right]\left[ {\left( {UY_{1} - LY_{1} - W_{j} } \right)\sigma + 1} \right]$$

And in the second case, the cell number where machine *j* is located is *c*$(c > 1)$ if:

47 $$CHG\;number > \mathop \sum \limits_{c = 1}^{C - 1} \left[ {\left( {UX_{c} - LX_{c} - L_{j} } \right)\sigma + 1} \right]\left[ {\left( {UY_{c} - LY_{c} - W_{j} } \right)\sigma + 1} \right]$$

Similarly, there are two cases to calculate the cell number where machine *j* is located based on CH number of selected CHG for machine *j.* In the first case, the cell number where machine *j* is located is one $\left( {c = 1} \right)$ if:

48 $$CH\;number \le \left( {UX_{1} - LX_{1} } \right)\left( {UY_{1} - LY_{1} } \right)\sigma^{2}$$

And in the second case, the cell number where machine *j* is located is *c*$(c > 1)$ if:

49 $$CH\;number > \mathop \sum \limits_{C = 1}^{C - 1} \left( {UX_{c} - LX_{c} } \right)\left( {UY_{c} - LY_{c} } \right)\sigma^{2}$$

The *MH* number calculation based on *CHG* number and cell number $c$ is done as follows:

50 $$\left\{ {\begin{array}{*{20}l} {MH\;number = L_{j} \sigma \left( {CHG\;number - 1} \right) + 1\quad if\;c = 1 \quad and \quad L_{j} = UX_{c} - LX_{c} } \hfill \\ {MH\;number = CHG\;number + \left[ {\frac{CHG\;number}{{(UX_{c} - LX_{c} - L_{j} )\sigma + 1}} - e} \right]\left( {L_{j} \sigma - 1} \right)\quad if\;c = 1 \quad and \quad L_{j} \ne UX_{c} - LX_{c} } \hfill \\ \end{array} } \right.$$

51 $$\left\{\begin{array}{ll} NOSC = CHG\;number - \mathop \sum \limits_{c = 1}^{C - 1} \left[ {\left( {UX_{c} - LX_{c} - L_{j} } \right)\sigma + 1} \right]\left[ {\left( {UY_{c} - LY_{c} - W_{j} } \right)\sigma + 1} \right]\quad if \quad c > 1 \\ MH\;number = L_{j} \sigma \left( {NOSC - 1} \right) + TCH_{c - 1} + 1\quad if c > 1 \quad and \quad L_{j} = UX_{c} - LX_{c} \\ MH\;number = NOSC + \left[ {\frac{NOSC}{{(UX_{c} - LX_{c} - L_{j} )\sigma + 1}} - e} \right]\left( {L_{j} \sigma - 1} \right) + TCH_{c - 1} \quad if \quad c > 1 \quad and \quad L_{j} \ne UX_{c} - LX_{c} \end{array}\right.$$

where $e$ is a very small number and sign [ ] represents a floor function.

The calculation of *CH* numbers of each *CHG* based on the *MH* number and cell number would be as follows if it is defined in set *H*:

52 $$H = \left\{ {\left. {MH\;number + k + \left( {UX_{c} - LX_{c} } \right)m\sigma } \right|\left( {k,m} \right):\quad \begin{array}{*{20}c} {k = 0 \quad to \quad k = L_{j} \sigma - 1} \\ {m = 0 \quad to \quad m = W_{j} \sigma - 1} \\ \end{array} } \right.$$

The horizontal and vertical coordinates of machine *j* are calculated based on the number *MH* and related occupied *CHG *as below.

53 $$\left\{ {\begin{array}{*{20}l} {\alpha_{j} = LX_{c} + \frac{MH\;number}{\sigma } - \left[ {\frac{MH\;number}{{\left( {UX_{c} - LX_{c} } \right)\sigma }}} \right]\left( {UX_{c} - LX_{c} } \right) + \frac{{L_{j} }}{2} - \frac{1}{\sigma }} \hfill \\ {\beta_{j} = LY_{c} + \frac{{\left[ {\frac{MH\;number}{{\left( {UX_{c} - LX_{c} } \right)\sigma }}} \right]}}{\sigma } + \frac{{W_{j} }}{2}} \hfill \\ \end{array} \quad if\;c = 1} \right.$$

54 $$\left\{ {\begin{array}{*{20}l} {\alpha_{j} = LX_{c} + \frac{{MH\;number - TCH_{c - 1} }}{\sigma } - \left[ {\frac{{MH\;number - TCH_{c - 1} }}{{\left( {UX_{c} - LX_{c} } \right)\sigma }}} \right]\left( {UX_{c} - LX_{c} } \right) + \frac{{L_{j} }}{2} - \frac{1}{\sigma }} \hfill \\ {\beta_{j} = LY_{c} + \frac{{\left[ {\frac{{MH\;number - TCH_{c - 1} }}{{\left( {UX_{c} - LX_{c} } \right)\sigma }}} \right]}}{\sigma } + \frac{{W_{j} }}{2}} \hfill \\ \end{array} \quad if\;c > 1} \right.$$

Where sign [ ] represents a floor function.

### The initial population

The next step after determination of chromosome structure is generating an initial population of chromosomes. In this section, the hierarchical procedure for filling the layout genes and scheduling genes of a chromosome is explained separately.

**Fig. 5:**
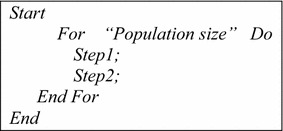
Pseudo code of filling the scheduling genes

At first, the filling procedure of scheduling genes with a corresponding pseudo code, shown in Fig. 5, is defined according to the following steps:

- *Step 1* The operations of all parts are permuted on machines randomly based on decimal numbers *(i.k)* in scheduling genes.
- *Step 2* A machine is chosen randomly among the machines that do the same operation based on Constraint (5). This means the information of the part and the operations that are related to it should appear only once in each chromosome.

**Fig. 6:**
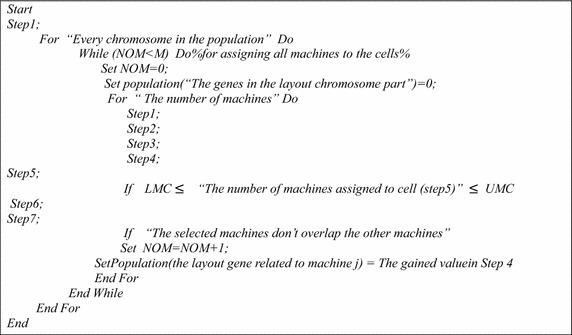
Pseudo code of filling the layout genes

Next, the filling procedure for layout genes with a corresponding pseudo code, shown in Fig. 6, is defined according to the following steps:

- *Step 1* The value for all CH’s is calculated according to the dimension of existing cells and multiplier $\upsigma$.
- *Step 2* One machine *j* is selected randomly from the unselected machines.
- *Step 3CHG’s* (i.e., series $S_{j}$) are calculated through formulas (44) and (45) for the selected machine *j*.
- *Step 4* A* CHG* from set $S_{j}$ is selected randomly and is placed in the layout gene.
- *Step 5* The cell number where machine *j* is located is obtained through formulas (46) and (47) based on the *CHG* number or through formulas (48) and (49) based on *CH* number of *CHG* selected for machine *j*.
- *Step 6* The MH number is obtained through formulas (50) and (51) based on the cell number and the *CHG* number that the machine *j* has occupied.
- *Step 7CH* numbers of selected *CHG* for the machine *j* is obtained through formula (52) based on the *MH* number and cell number.

### Calculation procedure of fitness function

In this section, the procedure of calculating objective function value is described. Since there are three different ingredients in the objective function, including makespan, tardiness penalty costs and inter-cell/intra-cell movements’ costs, they are calculated in three phases to evaluate the objective function for each chromosome.

**Fig. 7:**
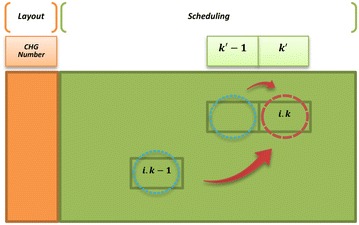
Evaluating makespan ingredient of objective function for a scheduling gene

In the first phase, for evaluating makespan ingredient of the objective function, it is needed some modifications on the chromosome to make the calculation of makespan possible. This is because by considering the constraints (7) and (8), the calculation of the completion time of each processing position *k′* of machine *j*$(CTM_{{k^{\prime}j}} )$ is possible provided that two conditions:

Firstly, the completion time of processing position $k^{\prime} - 1$ of machine *j* is calculated in the chromosome. Secondly, the completion time of operation $k - 1$ of part *i* should be gained in the chromosome if operation *k* of part *i* is processed in the processing position $k^{\prime}$ of machine *j* (Fig. 7).

Therefore, the following algorithm is presented for modifying permutation in scheduling section of each chromosome until it would be possible to evaluate the makespan in each chromosome.

- *Step 1* In the scheduling section of each chromosome, the genes for which it would be possible to evaluate their completion time are evaluated by considering two mentioned conditions.
- *Step 2* For each chromosome string, the first gene that its completion time has not been calculated yet is considered. Then, one of them is chosen randomly.
- *Step 3* the chromosome strength at was selected in step 2 is considered. Amongt he other genes that their completion time in that chromosome has not gained yet, the gene that has the minimum decimal number is chosen and is replaced with the selected gene in step 2. If there is more than one gene that has the minimum decimal number, one of them is chosen randomly and replaced with the selected gene in step 2.
- *Step 4* The first, second and third steps are repeated until the completion times of all genes $(CTM_{{k^{\prime}j}} )$ are obtained. Following these steps and considering the constraints (9) and (10), the $(CTP_{i} )$ values and the makespan value $(C_{\hbox{max} } )$ are returned.

**Fig. 8:**
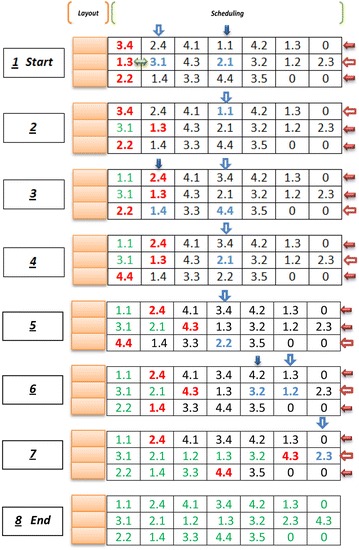
An example for calculation of fitness function

An example for calculation of fitness function is illustrated in Fig. 8, where there is a chromosome with three machines and four parts. In this example, the scheduling genes of the presented chromosome are reordered in eight steps. However, because of random selections in steps 2 and 3, the number of these steps could be more or less.

In the second phase, by considering the amounts of the layout genes of each chromosome (*CHG’s*) and formulas (43)–(54), the following values are obtained: the cell number, *MH* number, coordinates of each machine and the distances between machines.

In the third phase, the calculation of fitness function of each chromosome is gained by considering the objective function and the information obtained from the first and second phases.

### Selection

The selection function chooses the parents for the production of next generation. In the presented algorithm, a grading function for scaling is used that prevents of more extension of raw scores. In this scaling, the grade related to each individual (chromosome) is a rank that is assigned to each individual in a population after being sorted. Therefore, the grade related to the best score is 1, for the next is 2 and so forth. The ranking function will assign a rank to each individual as described below:

1. At first, all individuals of a population are ranked based on their fitness function value (i.e., objective function value) increasingly. An individual with rank *n* is given value $1 /\sqrt n$.
2. Next, total measure amounts of the whole population are equal with the number of needed parents for the production of the next generation.
3. The value $1 /\sqrt n$ is placed in the interval (0, 1] and correctness coefficient $(\alpha )$ is obtained from the below formula.

$$\alpha = \frac{Population\;size}{{\mathop \sum \nolimits_{n = 1}^{Population\;size} \frac{1}{\sqrt n }}}$$

By multiplying the value $1 /\sqrt n$ of each individual bycoefficient $(\alpha )$, a scaling number is given toeach individual which is used by roulette wheel rule for selecting parents

### Reproduction operators

Offsprings in each new generation are created using recombination operators (i.e., crossover and mutation) as described below:

#### Crossover

Crossover operator designed in this algorithm makes two offsprings from two selected parents by considering each chromosome string separately. To implement this operator, three crossover points are selected on each string. Clearly, the first crossover point is between the first and the second gene, and two other crossover points are selected after the first crossover point, randomly. This crossover acts in two phases as described below.

**Fig. 9:**
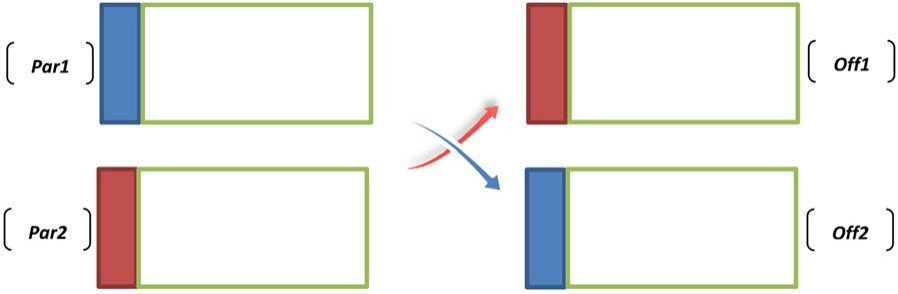
Crossover operator for the first gene

In the first phase, the first gene of the first offspring is copied from the first gene of the second parent and the first gene of the second offspring is copied from the first gene of the first parent. A simple example of how crossover operator works is shown in Fig. 9.

In the second phase, the crossover operator for scheduling genes of each chromosome acts distinctly from the layout gene. The length of each chromosome string may be different from other chromosome strings due to the fact that the process routings for part types can be flexible. Therefore, the steps of crossover operator in the scheduling section of each chromosome string are described as below:

- *Step 1* Along the length of the two parents, two crossover points are chosen in similar points, randomly. Then, the length of first offspring is calculated by the below formula:
  $$\begin{aligned} & {\text{length}}\;{\text{of}}\;{\text{chromosome}}\;{\text{string}}\;{\text{of}}\;{\text{the}}\;{\text{first}}\;{\text{offspring}} \\ & \quad = {\text{``distance}}\;{\text{between}}\;{\text{two}}\;{\text{random}}\;{\text{points''}}\, + \,{\text{``length}}\;{\text{of}}\;{\text{chromosome}}\;{\text{of}}\;{\text{the}}\;{\text{second}}\;{\text{parent''}}\, - \,\omega \\ \end{aligned}$$
  where $\omega$ is the number of genes that are common along the two chosen points of the first parent and the whole chromosome string of the second parent.
- *Step 2* The section between two random points of the first parentis copied into the first offspring.
- *Step 3* Starting from the second crossover point of the second parent, the other unused numbers in an order that they appear are copied in the second parent. If it reaches to the end of the string, it starts from the beginning and copies the remaining genes in order.
- *Step 4* In this step, the correction of the offsprings is made. The random choice of a machine among the machines that process the same operations based on constraint (5) means each part information and its related operations (*i,k*) should appear only once in each chromosome.
- *Step 5* The second offspring is produced in the same way by reversing the parents’ role.

**Fig. 10:**
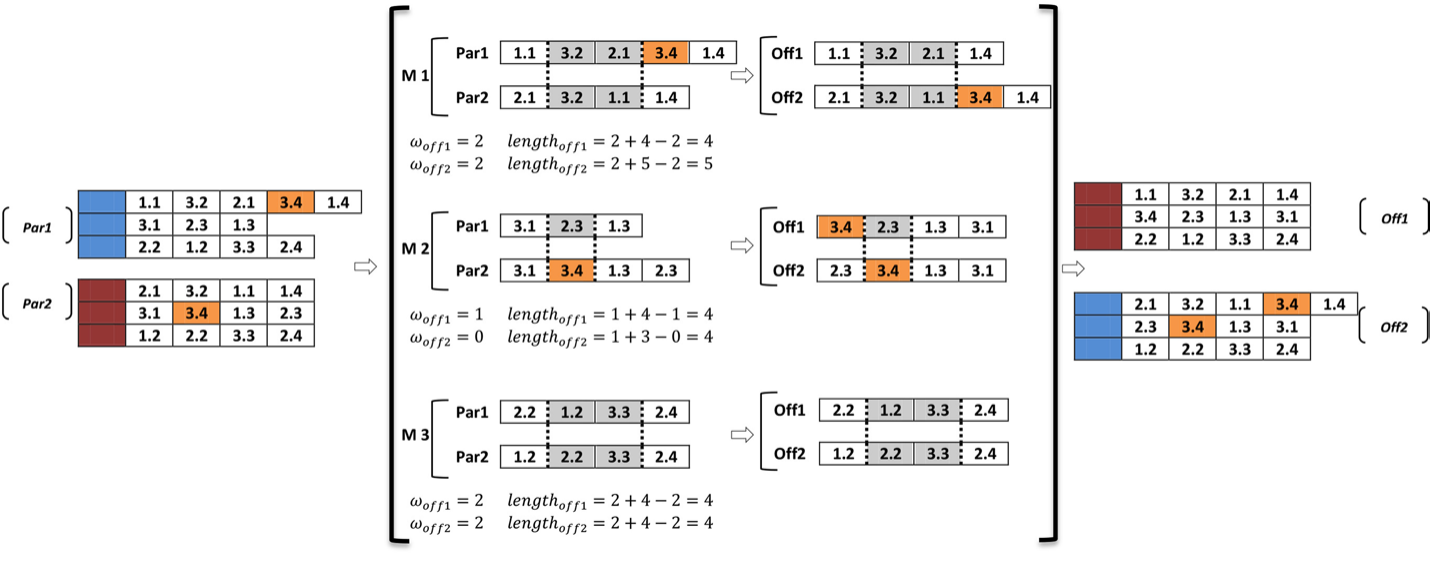
Crossover operator for scheduling genes

An example of implementing crossover operator for scheduling section is shown in Fig. 10.

**Fig. 11:**
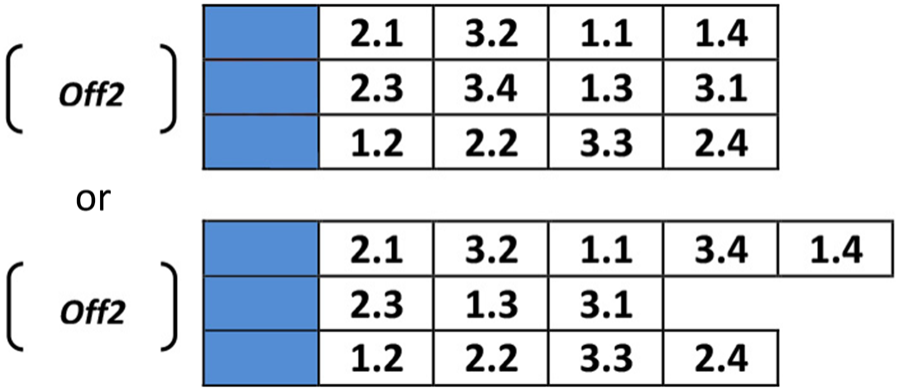
Repairing an offspring

As it is clear for *off2* in Fig. 10, the fourth operation of part 3 (i.e., 3.4) is processed by both machine 1 and machine 2. This makes the obtained offspring unfeasible. Hence, the chromosome should be repaired. For that reason, one of them is chosen randomly and the other is deleted from the chromosome (Fig. 11).

#### Mutation

Mutation operator in the layout and scheduling genes of each offspring operates separately by mutation probability $P_{m}$ and $P_{{m^{{\prime }} }}$ respectively, as described in the two following phases.

In the first phase, a random resetting mutation operator is done on the layout gene. A random number in the interval $\left( {0 1} \right]$ is generated and compared with $P_{m}$. If the random number is smaller than $P_{m}$, one of thechromosome strings will be selected by chance and an amount of allowable set $S_{j}$ will be selected in that situation and other amounts of different offspring genes will be changed in an order according to the steps that were explained in “The initial population” section, and new amounts will be replaced.

In the second phase, swap mutation operator is done on the scheduling genes. At first, a random number is produced for each chromosome string. Then, if the random number is smaller than $P_{{m^{{\prime }} }}$, two positions of genes will be randomly selected and their amounts will be replaced by each other.

### Termination criterion

Termination criterion in the proposed algorithm is the number of iterations. Therefore, the production of new generations will be continued until the number of iterations is reached. That value depends on the problem size.

## Experimental results

### Two illustrative numerical examples

In this section, two experiments are performed to validate the proposed model and compare the effects of the sequential and concurrent approach in the CM design.

To verify the proposed model and reveal that the concurrent integration of parts scheduling with CF and machines layout is more effective than sequential approach, two small-sized examples are solved by a Branch and Bound (B&B) method under Lingo 8.0 software on an Intel(R) core(TM) i5 CPU 2.6 GHz personal computer with 4.00 GB RAM.

In the sequential approach, the proposed model is solved to find the optimal values of decision variables for CF and CL. Then, using the obtained solution the optimal solution of the scheduling problem is determined. To implement this approach, the main model is solved by excluding the components (1.1) and (1.2) associating with makespan and tardiness from the objective function in the first step. Then, in the second step, the main model is solved by regarding the values obtained for decision variables of CF and CL as input parameters and excluding the component (1.3) associating with material handling costs from the objective function.

In the concurrent approach, the proposed model is optimally solved including all three components in the objective function to find the optimal solution of integrating parts scheduling with CF and machines layout simultaneously. Finally, the objective function values (OFV) obtained for the optimal solutions of both approaches are compared.

In these two numerical examples, the data is randomly generated which includes process routing for each part type and processing time for each operation. Machines are selected randomly for each process routing and the processing times of operations are random integer numbers between 2 and 30. The machines should be assigned to two cells. The due date for all part types is 40 min. The material handling time for all part types per distance unit is 3 min. The inter-cell and intra-cell material handling costs for all part types per distance unit are 5$ and 2$, respectively. The tardiness penalty for all part types per time unit is 3$. The factory costs per time unit are 25$.

 Tables 2 and 3 represent parts’ route sheet with the processing time of each operation of each part type on each machine for the first and second example, respectively.

**Table 2:** Processing times of part operations for the first example

Machines	P1		P2		P3		P4	
	1	2	1	2	1	2	1	2
M1	18			15	3			11
M2	24		5			10	6	
M3		5	19	8			20

**Table 3:** Processing times of part operations for the second example

Machine	P1			P2			P3			P4		
	1	2	3	1	2	3	1	2	3	1	2	3
M1	7				5			25		17		
M2			29	14				30				3
M3		25				28	12					8
M4	3					19			8		11	

 Tables 4 and 5 demonstrate machines dimensions and coordinates of cell partitions for the first and second example, respectively.

**Table 4:** Machines dimensions and coordinates of cells partitions for the first example

Parameter	Machines’ information		Cells’ information				
	$L_{j}$	$W_{j}$		$LX_{c}$	$UX_{c}$	$LY_{c}$	$\varvec{UY}_{\varvec{c}}$
M1	4	2	C1	3	8	3	13
M2	2	3	C2	11	17	5	11
M3	3	5

**Table 5:** Machines dimensions and coordinates of cells partitions for the second example

Parameter	Machines’ information		Cells’ information				
	$L_{j}$	$W_{j}$		$LX_{c}$	$UX_{c}$	$LY_{c}$	$\varvec{UY}_{\varvec{c}}$
M1	4	2	C1	2	7	2	10
M2	2	3	C2	10	15	6	14
M3	5	3					
M4	2	2					

**Fig. 12:**
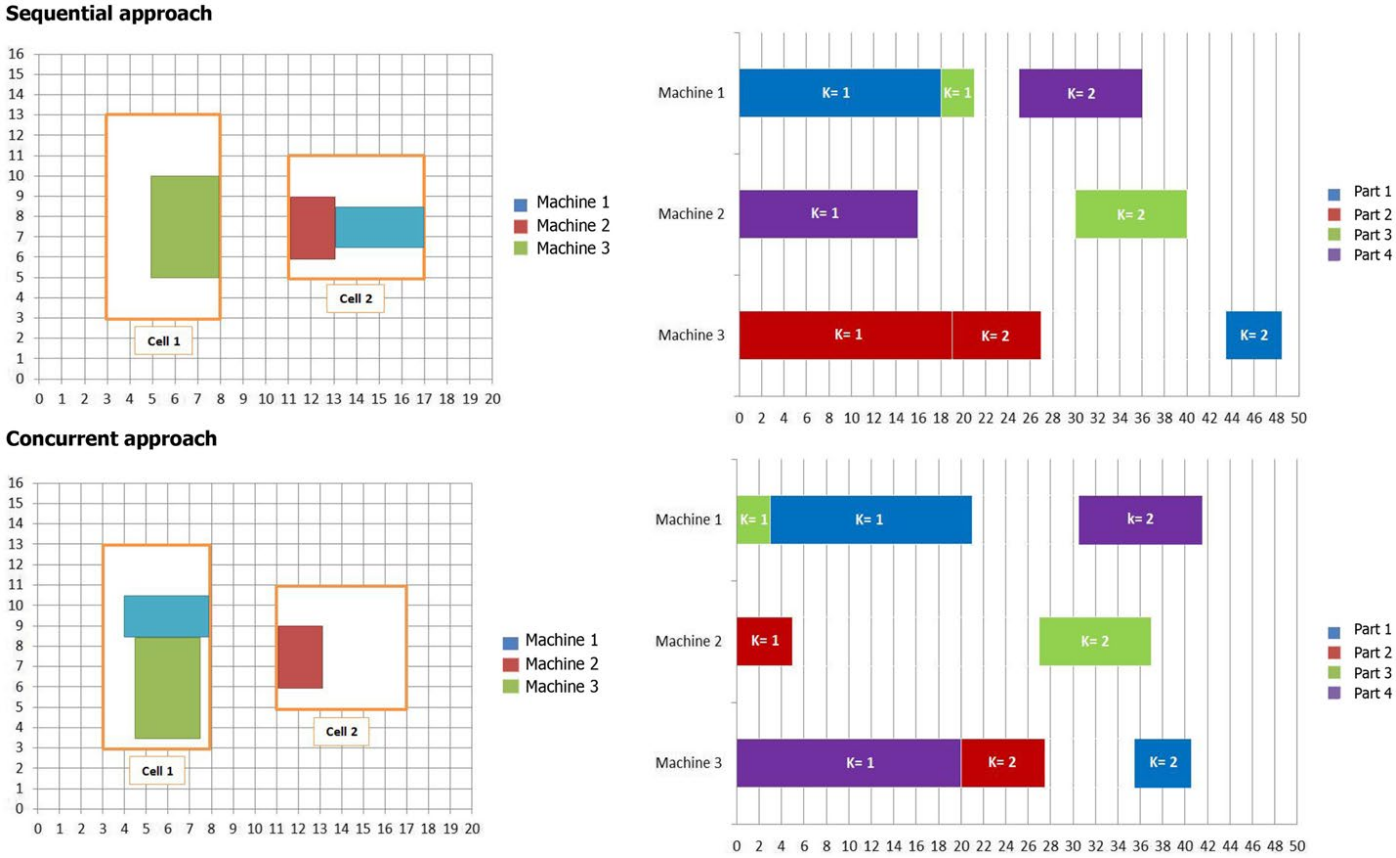
Intra-cell layout and Gantt chart of part operations scheduling for the first example

**Fig. 13:**
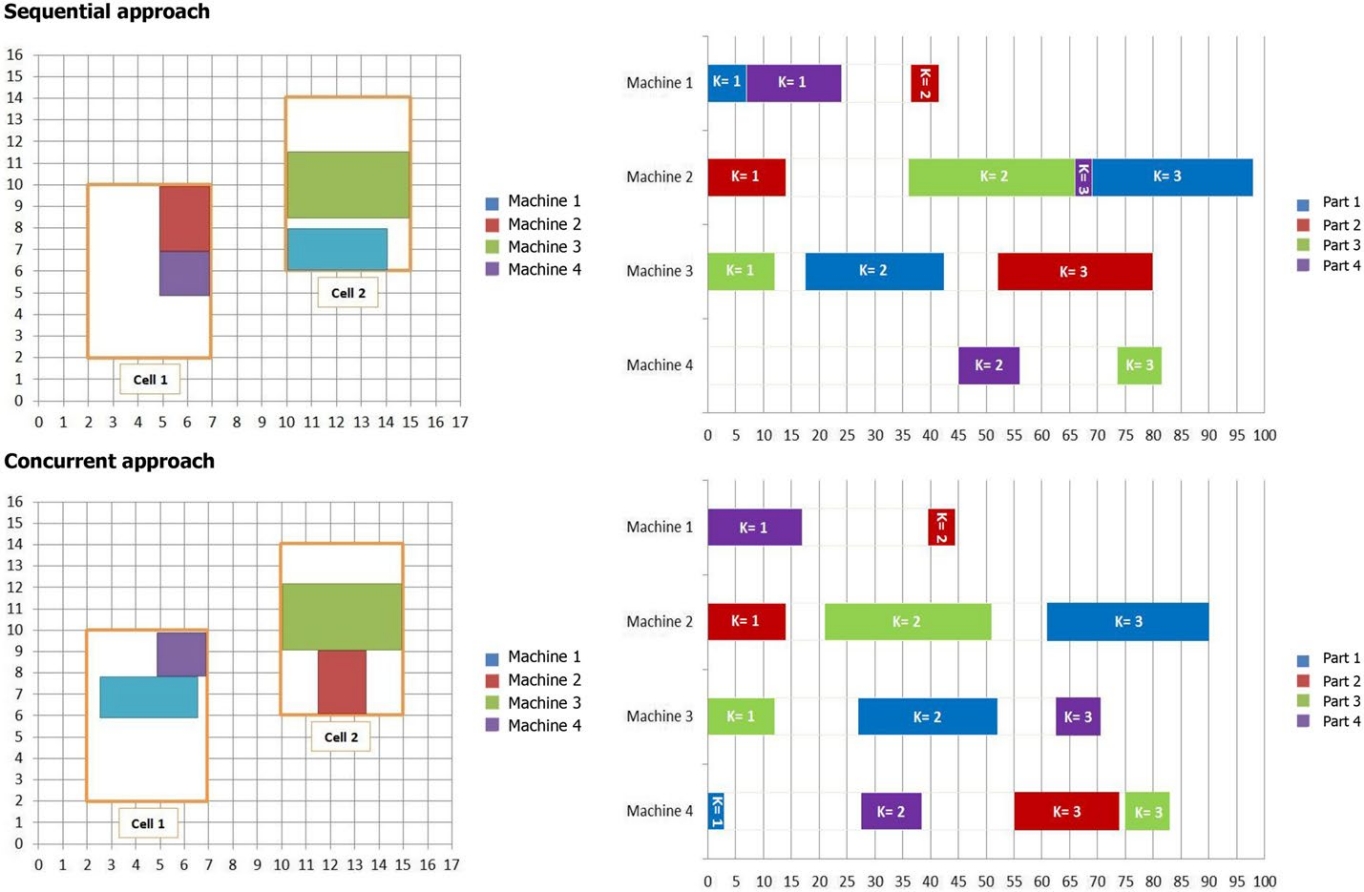
Intra-cell layout and Gantt chart of part operations scheduling for the second example

Figures 12 and 13 illustrate the location of each machine in each cell for the sequential and concurrent approach of examples 1 and 2, separately. Also, Gantt charts of part operations scheduling have been projected in these figures. A Gantt chart illustrates the sequence of operations of part types processed on machines along with processing time and material handling time. It is depicted for the sequential and concurrent approach of each example, separately.

Now, the solution obtained for the first example is discussed. This example consists of four part types and three machine types. Each part type requires two operations that each one can be processed by only one machine type selected from two alternative machine types with different processing times. For instance, there are two process routings for part type 1, the first operation of part type 1 can be processed either on machine type 1 or machine type 2 while the second one can be done only on machine type 3. Also, there are four, one and two process routings for part types 2, 3 and 4, respectively.

The optimal OFVs obtained for the proposed model in two sequential and concurrent approaches are shown in Table 6 for the first example.

**Table 6:** Objective function value and its cost components for the first example

Cost	Value		
	Sequential approach	Concurrent approach	Improvement by concurrent approach
Factory costs × *C*_max_	25 × 48.5 = 1212.5	25 × 41.5 = 1037.5	175 (16.7 %)
Tardiness penalty	25.5	6	19.5 (325 %)
Material handling	54.5	91.5	−37 (67.9 %)
Total (OFV)	1292.5	1135	157.5 (13.9 %)

Since in the sequential approach, the objective function is optimized without components related to makespan and tardiness, the model is able to find the optimal value for material handling cost equal to 54.5. However, when the components related to makespan and tardiness are included in the objective function in the concurrent approach, the previous optimal material handling cost increase due to the effect of scheduling parts on machines layout. Switching from sequential approach to concurrent approach reduces the value of makespan from 48.5 to 41.4 and the value of tardiness penalty from 25.5 to 6, which are remarkable improvements for these components. On the other hand, incorporating parts scheduling in the concurrent approach increases the value of material handling cost from 54.5 to 91.5. Totally, OFV is improved about 14 % by switching from sequential approach to concurrent approach. This achievement was expectative since simultaneous decisions making about interrelated decisions machines layout and parts scheduling brings the capability for the model to optimize all components of the objective function as an optimal strategy in designing a CMS.

Next, the second example consists of four part types and three machine types. Each part type requires three operations that each one can be performed by at most two machine types. There are two process routings for each part type.

The optimal OFVs obtained for the proposed model in two sequential and concurrent approaches are shown in Table 7 for the second example.

**Table 7:** Objective function value and its cost components for the second example

Cost	Value		
	Sequential approach	Concurrent approach	Improvement by concurrent approach
Factory costs × *C*_max_	25 × 98 = 2450	25 × 90 = 2250	200 (8.9 %)
Tardiness penalty	505.5	472.5	33 (7 %)
Material handling	176.5	188.5	−12 (6.8 %)
Total (OFV)	3132	2911	221 (7.6 %)

A similar improvement in the obtained OFV is observed for the second example in Table 7 as it was expected due to the advantage of concurrent approach.

As can be seen in Figs. 12 and 13, when makespan and tardiness components are included in the objective function in the concurrent approach, cell formation, machines layout, and parts scheduling change completely in comparison with sequential approach. It can be concluded that even if factory cost is very high forcing to complete all parts as soon as possible, one should group machines, locate them and schedule operations simultaneously.

To conclude, by comparing the OFVs obtained for examples 1 and 2 in the sequential and concurrent approaches, it is recognized that if cells are configured, machines are located and operations are scheduled sequentially, the optimum strategy with the minimum costs cannot be reached.

### Evaluation of the proposed GA

In order to evaluate the performance of the GA in comparison with B&B method for the sequential and concurrent approaches, ten instances have been generated with the random data by inspiration from the literature. The GA has been coded in MATLAB software and run ten replicates on an Intel(R) core(TM) i5 CPU 2.6 GHz computer with 4.00 GB RAM for each test problem, and the best, as well as the average of the obtained solutions in ten runs, have been reported in Table 8. Also, for all test problems, the linearized programming model has been solved by a B&B method under LINGO software. In large-sized problems, the LINGO program has been interrupted after 12 h, and the best-obtained solution has been reported. Table 8 illustrates the information of test problems and the obtained solutions with the computational times by GA and B&B method as an exact method for the sequential and concurrent approaches.

**Table 8:** Comparison of GA solutions and exact solutions

Problem no.	No. of machines	No. of parts	No. of operations per part	No. of processing positions per machine	No. of cells	Sequential approach					Solution GAP
						B&B		GA			
						OFV	Time (s)	Best OFV	Average OFV	Time (s)	
1	3	4	2	4	2	1292.5*	16	1292.5	1292.5	1.6	–
2	4	4	3	3	2	3474.5*	276	3474.5	3474.5	3.9	–
3	4	4	3	4	2	3132*	20,350	3132	3132	14.6	–
4	4	5	3	4	2	3688.5*	38,405	3688.5	3700.5	15.4	–
5	5	6	3	4	2	5010.5	43,200	4798	4851.7	18.5	−3.3
6	6	8	3	4	2	6490.7	43,200	6206.5	6456.5	23.3	−4.6
7	8	10	4	5	3	13,728.6	43,200	12,802	13,588.4	33.8	−7.2
8	10	12	5	6	3	22,347.4	43,200	20,997	21,190.8	42.4	−6.4
9	15	25	3	5	4	NA	43,200	30,677	30,152.7	61.1	NA
10	20	40		6	6	NA	43,200	86,110	77,090.3	102.5	NA

Problem no.	No. of machines	No. of parts	No. of operations per part	No. of processing positions per machine	No. of cells	Concurrent approach					Solution GAP	OFV Improvement B&B (%)	OFV Improvement GA (%)
						B&B		GA					
						OFV	Time (s)	Best OFV	Average OFV	Time (s)			
1	3	4	2	4	2	1135*	185	1135	1112.5	2.9	–	13.9	13.9
2	4	4	3	3	2	3089*	1203	3089	3089	7.6	–	12.5	12.5
3	4	4	3	4	2	2911*	29,900	2911	2911	18.2	–	7.6	7.6
4	4	5	3	4	2	3557.3	43,200	3520	3691.7	19.2	−1.1	3.7	4.8
5	5	6	3	4	2	4556.7	43,200	4323.5	4482.7	23.3	−5.4	10	11
6	6	8	3	4	2	5764.2	43,200	5486	5543.9	31.4	−5.1	12.6	13.1
7	8	10	4	5	3	10,582.5	43,200	9957.5	10,233.8	56.7	−6.3	29.7	28.6
8	10	12	5	6	3	18,632.4	43,200	17,453	18,255.6	68.47	−6.8	19.9	20.3
9	15	25	3	5	4	NA	43,200	23,543	24,040.3	108.3	NA	NA	30.3
10	20	40		6	6	NA	43,200	67,872	68,253.6	221.1	NA	NA	26.9

* Optimal solution

As it can be seen from Table 8, GA has found optimal solutions for problems 1–4 for sequential approach and optimal solutions for problems 1–3 for the concurrent approach in which optimal solutions are obtained by B&B method. No feasible solution is found by B&B for problems 9 and 10 in both approaches due to the complexity of the model. Furthermore, for the rest of problems in both approaches, the best solutions found by GA are better than the solutions found by B&B in much less time. On average, the comparisons between the OFVs obtained by sequential and concurrent approaches indicate that the OFV improvement is around 17 % by GA and 14 % by B&B.

These promising results obtained by the proposed GA prove the efficiency of the designed algorithm enhanced by the matrix-based chromosome structure. Furthermore, the developed GA is elaborately designed to create feasible solutions by using some defined formulas, efficient crossover and mutation operators and established procedures calculating fitness function and generating initial population.

## Conclusion

In this article, a novel integrated mathematical model has been formulated for designing a cellular manufacturing system considering three problems simultaneously: cell formation, intra-cell layout, and parts scheduling. The results show that considering these three significant decisions in a simultaneous manner contributes to a successful CM implementation in the manufacturing environment. All these problems have been optimized due to three components in the objective function including makespan time, tardiness penalty, and inter-cell and intra-cell material handling cost. By investigating two integration approaches, namely sequential and concurrent, it was revealed that to reach an optimal solution, all stages of CMS (CF, machine layout, and part scheduling) must be designed simultaneously. It In sequential approach, since cells are configured at first in order to decrease inter-cell and intra-cell movements costs, and finally operations are scheduled in order to optimize time factors in the objective function consisting of tardiness and makespan, the global optimal solution is not reached, although it is attainable in a concurrent approach. The advantages of the proposed model were as follows: designing layout of unequal-area machines in cells with continuous space, introducing alternative process routings for parts with different operation sequence, exerting the effects of distances between locations or cells on part scheduling, becoming both cost and time of part movements dependent on (1) traveled distance, (2) part type and (3) movement type (inter-cell and intra-cell), computing the completion time of each part by considering: (1) movement times, (2) processing times and (3) waiting times, and finally, determining three interrelated designing stages (i.e., CF, machines layout, and parts scheduling) in designing a CMS simultaneously. For transforming mixed-integer nonlinear programming formulation into a mixed-integer linear counterpart, some linearization techniques have been proposed. In order to verify the performance of the proposed model, two numerical examples have been solved. It was revealed that concurrent approach surpasses sequential approach in improving the quality of the obtained solutions in the CM system design.

Because of the complexity of the proposed model, Lingo software cannot obtain the optimum solution for medium or large-sized problems. Hence, a genetic algorithm has been developed that its excellent advantages were as follows: determining the exact location of machines in continuous-area cells; computing exact completion time of each operation of each part type, proposing heuristic crossover and mutation operator. The obtained results show that the best solutions found by GA are better than those found by Lingo in much less time especially as the size of problem increases. Incorporating other features, such as uncertainty in part demands, machine time capacity and cost coefficients, integrating with reliability and labor issues and considering dynamic issues will be left to future research.
